# Supplementary figures and images for: Resinous included phloem as a key indicator of authentic or fake agarwood
Source: PLoS One. 2024 Dec 2;19(12):e0312102. doi: 10.1371/journal.pone.0312102 (PMC11611195; doi:10.1371/journal.pone.0312102)

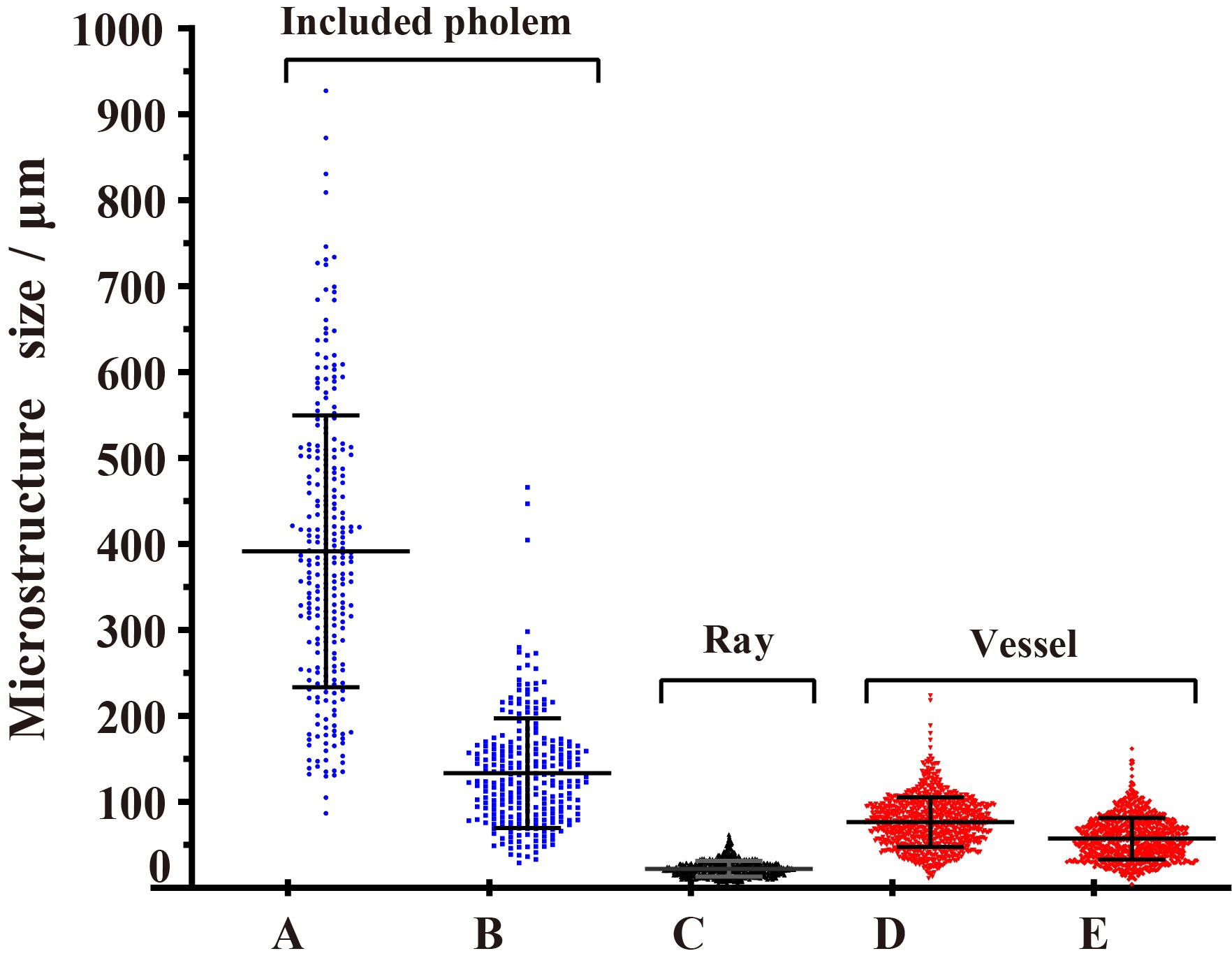

Supplement: S1 Fig — A and B: the tangential and radial widths of the included phloem; C: ray width; D and E: the tangential and radial widths of the vessel. (TIF) [file pone.0312102.s001.tif]
