# Supplementary material for: Resinous included phloem as a key indicator of authentic or fake agarwood
Source: PLoS One. 2024 Dec 2;19(12):e0312102. doi: 10.1371/journal.pone.0312102 (PMC11611195; doi:10.1371/journal.pone.0312102)
Supplement: S1 Table — # Wild agarwood, ## Artificially induced agarwood.; a) aqueous extract solution of agarwood (AQE), b) alcoholic extract solution of agarwood (ALE), c) supercritical extract solution of agarwood (SUE), d) alcoholic extract solution with high resin content Chi-Nan agarwood (ALEH). (DOCX) [file pone.0312102.s002.docx]

**Supporting information**

**S1 Table. Information on the samples used in this study.**

| **No.** | **Trade name** | **Genus/Species** | **Collected location** | **Type** |
| --- | --- | --- | --- | --- |
| **C01** | Bǎntóu agarwood ^#^ | *A. sinensis* | Haikou, China | The agarwood came from various producing areas. |
| **C02** | Agar-wit agarwood ^##^ | *A. sinensis* | Huazhou, China |  |
| **C03** | huǒlào agarwood ^##^ | *A. sinensis* | Maoming, China |  |
| **C04** | Chònglóu agarwood ^#^ | *A. sinensis* | Dingan, China |  |
| **C05** | píyǒu agarwood ^#^ | *A. sinensis* | Haikou, China |  |
| **C06** | kězǐ agarwood ^#^ | *A. crassna* | Hongkong, China |  |
| **C07** | Agar-wit agarwood ^##^ | *A. agallocha* | Bangladesh |  |
| **C08** | Wood chip ^##^ | *A..subintegra* | Laos |  |
| **C09** | Agar-wit agarwood ^##^ | *A. crassna* | Cambodia |  |
| **C10** | Wood chip | *A. crassna* | Vietnam |  |
| **C11** | Semi-submerged wood block | *Aquilaria* or *Gyrinops* | Sumatrina |  |
| **C12** | Wood block | *Aquilaria* or *Gyrinops* | Kalimantan |  |
| **C13** | Wood block | *Aquilaria* or *Gyrinops* | Papua New Guinea |  |
| **C14** | Special grade wood block | *A. filaria* | Ilian, Indonesia |  |
| **C15** | Wood chip | *A. malaccensis* | Malaysia |  |
| **C16** | Agar-wit agarwood ^##^ | *A. sinensis* | Huazhou, China |  |
| **C17** | Agar-wit agarwood ^##^ | *A. sinensis* | Haikou, China |  |
| **C18** | Wood chip | *A. yunnanensis* | Xishuangbanna, China |  |
| **C19** | Wood chip | *A. sinensis* | Zhangzhou, China |  |
| **C20** | Wood chip | *A. sinensis* | Qingshui, China |  |
| **C21** | Resin-free agarwood wood | *A. sinensis* | Haikou, China | The Laboratory made fake agarwood |
| **C22** | Ordinary agarwood A | *A. sinensis* | Haikou, China |  |
| **C23** | Ordinary agarwood B | *A. sinensis* | Haikou, China |  |
| **C24** | Resin-free agarwood wood 01 ^a)^ | *A. sinensis* | Haikou, China |  |
| **C25** | Resin-free agarwood wood 02 ^b)^ | *A. sinensis* | Haikou, China |  |
| **C26** | Resin-free agarwood wood 03 ^c)^ | *A. sinensis* | Haikou, China |  |
| **C27** | Resin-free agarwood wood 04 ^d)^ | *A. sinensis* | Haikou, China |  |
| **C28** | Ordinary agarwood A01 ^a)^ | *A. sinensis* | Haikou, China |  |
| **C29** | Ordinary agarwood A02 ^b)^ | *A. sinensis* | Haikou, China |  |
| **C30** | Ordinary agarwood A03 ^c)^ | *A. sinensis* | Haikou, China |  |
| **C31** | Ordinary agarwood A04 ^d)^ | *A. sinensis* | Haikou, China |  |
| **C32** | Ordinary agarwood B01 ^a)^ | *A. sinensis* | Haikou, China |  |
| **C33** | Ordinary agarwood B02 ^b)^ | *A. sinensis* | Haikou, China |  |
| **C34** | Ordinary agarwood B03 ^c)^ | *A. sinensis* | Haikou, China |  |
| **C35** | Ordinary agarwood B04 ^d)^ | *A. sinensis* | Haikou, China |  |
| **C36** | Rǔhú Chi-Nan agarwood | *A. sinensis* | Hannan, China | The uncertain agarwood from the market |
| **C37** | Tángjié Chi-Nan agarwood | *A. sinensis* | Hannan, China |  |
| **C38** | Aōshēn Chi-Nan agarwood | *A. sinensis* | Hannan, China |  |
| **C39** | Wood block | Unknown | Haikou, China |  |
| **C40** | Wood batten | Unknown | Bozhou, China |  |
| **C41** | Wood block | Unknown | Hehuachi, China |  |
| **C42** | Wood block | Unknown | Hehuachi, China |  |
| **C43** | Bracelet | Unknown | Vietnam |  |
| **C44** | Wood block | Unknown | Haikou, China |  |
| **C45** | Wood block | Unknown | Haikou, China |  |
| **C46** | Wood block | Unknown | Haikou, China |  |
| **C47** | Wood block | Unknown | United arab Emirates |  |
| **C48** | Wood chip | Unknown | Wanning, China |  |
| **C49** | Wood chip | Unknown | Haikou, China |  |
| **C50** | Wood block | Unknown | Dingan, China |  |
| **C51** | Wood block | Unknown | Bozhou, China |  |
| **C52** | Wood block | Unknown | Yulin, China |  |
| **C53** | Wood block | Unknown | Guangdong, China |  |

Note: ^#^ Wild agarwood, ^##^ Artificially induced agarwood.; a) aqueous extract solution of agarwood (AQE), b) alcoholic extract solution of agarwood (ALE), c) supercritical extract solution of agarwood (SUE), d) alcoholic extract solution with high resin content Chi-Nan agarwood (ALEH).
