# Supplementary material for: Resinous included phloem as a key indicator of authentic or fake agarwood
Source: PLoS One. 2024 Dec 2;19(12):e0312102. doi: 10.1371/journal.pone.0312102 (PMC11611195; doi:10.1371/journal.pone.0312102)
Supplement: Attachment — Submitted filename: Reviewers Comments Article-240128.docx [file pone.0312102.s003.docx]

**The presence of resinous included phloem indicative of genuine or fake agarwood**

**Comments**

This work describes the method for identifying authentic agarwood. Agarwood being a highly valued traditional medicine material and spice, is a lucrative commodity, leading to the prevalence of counterfeit material flooding the market. The paper describes a method to accurately distinguish between genuine agarwood and counterfeit products, a pressing concern of the industry. The method proposed is a simple and rapid assessment, crucial for customs enforcement agencies, and regulatory bodies like CITES.

Comments:

**Title: Could be modified as**

1. Assessing Authenticity in Agarwood through the Presence of Resinous Included Phloem
2. Resinous Included Phloem as a Key Indicator of fake or authentic Agarwood

**Introduction:**

1. Language needs improvement.
2. A logical flow is lacking in the introduction. It fails to introduce the need for identifying included phloem characterisitics to establish a reliable method for identifying authentic agarwood.

**Materials and Methods:**

1. Clarify the terms "uncertain agarwood", " producing areas " to ensure it's easily understood by readers.
2. What does ‘mainly referring to the common counterfeiting methods in the market’ mean?
3. When you mention 53 batches, how many samples were present in each batch? Or do you mean 53 samples?
4. Ensure consistency in terminology. Retain either batch or sample.
5. How many observations were recorded per sample?
6. What statistical analysis was employed?
7. Overall, the essential details of the sample collection process is conveyed, but breaking down the information and providing clarifications can enhance readability and comprehension.

**Results:**

1. The results effectively communicate the findings, but there are areas where clarity and conciseness could be improved.

**Discussion**

1. The readability of the discussion is moderate. While the information presented is clear in most parts, there are areas where the complexity of the subject matter or the improper usage of grammar poses a challenge.
2. With minor revisions to simplify the language and complex concepts, the readability of the report could be improved.
3. Consider rephrasing or condensing repetitive information to avoid redundancy.

**General observations:**

1. The language needs improvement.
2. The supplementary information can form a part of the main text.
3. Data related to the anatomical characteristics and be provided as supplementary information.
