# Supplementary material for: Resinous included phloem as a key indicator of authentic or fake agarwood
Source: PLoS One. 2024 Dec 2;19(12):e0312102. doi: 10.1371/journal.pone.0312102 (PMC11611195; doi:10.1371/journal.pone.0312102)
Supplement: Attachment — Submitted filename: Article-240128 with comments.docx [file pone.0312102.s004.docx]

**The presence of resinous included phloem indicative of genuine or fake agarwood**

Jian Feng^1^, Yangyang Liu^1*^, Peiwei Liu^1^, Yun Yang^1^, Anzhen Xie^1^, Jianhe Wei^1,2*^

^1^Hainan Provincial Key Laboratory of Resources Conservation and Development of Southern Medicine & International Joint Research Center for Quality of Traditional Chinese Medicine & Key Laboratory of State Administration of Traditional Chinese Medicine for Agarwood Sustainable Utilization, Hainan Branch of the Institute of Medicinal Plant Development, Chinese Academy of Medical Sciences and Peking Union Medical College, Haikou 570311, China.

^2^Key Laboratory of Bioactive Substances and Resources Utilization of Chinese Herbal Medicine, Ministry of Education & National Engineering Laboratory for Breeding of Endangered Medicinal Materials, Institute of Medicinal Plant Development, Chinese Academy of Medical Sciences and Peking Union Medical College, Beijing 100193, China.

*** Correspondence**

E-mail: yyliu@implad.ac.cn (YYL)

E-mail: wjianh@263.net (JHW)

**Abstract**

Agarwood, the resinous wood of the genus *Aquilaria* Lam. and *Gyrinops* Gaertn. of the family Thymelaeaceae, is a very valuable traditional medicinal material and spice. Due to the high economic value of agarwood and the pursuit of high profits by unscrupulous traders, which leads to the prevalence of counterfeit agarwood in the trade market, it is crucial to excavate the characterization of agarwood and establish a simple and rapid method of identifying authenticity. In this study, by comparing the authenticity of the microstructures of agarwood from different origins and artificially simulated fake agarwood, it was found that the included phloem of genuine agarwood was filled with brown to brownish resin, The resin was also deposited in the rays, naturally distributed, and stably present. While fake agarwood is divided into two categories, one itself is wood confusion (without included phloem), the other is artificially counterfeiting. However, the filling of resin in the included phloem of agarwood did not occur after artificially simulated counterfeiting treatments. Ten of the 18 batches of commercially available samples had the same microstructure of agarwood, and the included phloem of six of these samples was not filled with resin. Therefore, the resinous included phloem is a key characteristic structure of agarwood that can be used as a basis for authenticity identification. This will provide a convenient and rapid method for promoting and popularising agarwood authentication in trade, customs enforcement, CITES management, and other fields.

**Introduction**

Agarwood is the resinous wood of the genera *Aquilaria* Lam. and *Gyrinops* Gaertn. of the family Thymelaeaceae. It has been called different names in different cultures of the world, such as ‘Chēngxiáng’ in China, ‘gaharu’ in Indonesia and Malaysia, ‘agar’ in India, ‘oud’ in the Middle East, and ‘jin-koh’ in Japan [1-2]. Agarwood has been used as a traditional medicine for nearly two thousand years [3] to treat joint pain and is an anti-inflammatory, cardioprotective, sedative, and anticancer agent [4-6]. Agarwood is also a valuable spice with a wide range of applications in fragrances, perfumes, and religion. For example, the volatile oil of agarwood is used as a scent fixative in top-quality perfumes, smoked using agarwood such as agarwood threads, and processed into artefacts such as bead strings [7-8]. The natural distribution area of agarwood includes China, India, Laos, Cambodia, Thailand, Vietnam, Bangladesh, Indonesia, and Papua New Guinea [3,7,9]. All species of *Aquilaria* and *Gyrinops* genera have been listed in Appendix II of the Convention on International Trade in Endangered Species of Wild Fauna and Flora (CITES) since 2004 [10]. Therefore, the agarwood trade has been closely monitored by international regulators to ensure that such activities do not continue to damage the continued survival of these species in the wild.

The volume of agarwood traded in the international agarwood trading market reaches several hundred tons each year, with a turnover of tens of millions of dollars. The end market is mainly concentrated in Asia and the Middle East [11]. A healthy agarwood tree must be physically, chemically, and microbiologically harmed to gradually form agarwood, but the growth is slow and yields low [12-15]. With high market demand and high profits, unscrupulous traders carry out counterfeiting and adulteration through a variety of means, such as high-pressure oil injection, soaking spices to pretend to be high-quality agarwood, and substituting woods as agarwood: this phenomenon is more pronounced when they are processed into handicrafts (beads, bracelets, and statues) [16-17]. At present, research on agarwood mainly focuses on the formation mechanism of agarwood [18-19], species identification [20], the separation of sesquiterpenes and 2-(2-phenylethyl) chromones [21], and the development of modern chromatography and mass spectrometry analysis methods [22-24], and pharmacology [25-26]. Although some of the developed methods and standards for the analysis of agarwood quality meet the quality analysis and accurate identification of agarwood [27], they are not suitable for rapid on-site testing of agarwood because of the shortcomings of the large number of samples required, expensive equipment, specialized technicians, and long timeliness.

Light microscopy is the most widely used method for botanical and timber identification, and IAWA has established a classic database of wood anatomy [28-30]. Studies have been conducted to report the structure of the wood of *Aquilaria sinensis* Lour. Spreng, *Aquilaria malaccensis* Lam., and *Aquilaria yunnanensis* S.C. Huang [11,31-32]. Liu et al. proposed that the included phloem, also known as interxylary phloem and rays are the main sites of resin formation in *A.sinensis* [33-34]. The agar-wit technique was used to induce agarwood formation in *A. sinensis*, and the included phloem was filled with resin after 1 month [35]. An understanding of the anatomical structure of agarwood would enable establishing a simple and rapid method for authenticity identification. Therefore, this study will further verify whether the resinous included phloem, an important characteristic structure of agarwood, can be used as a basis for authentication. This will provide a convenient and rapid method for promoting and popularising agarwood authentication in trade, customs enforcement, and CITES management.

**Materials and methods**

**Materials collection**

A total of 53 batches of samples were collected in this study (Table 1). 20 batches of agarwood samples (C1-C20) from 10 producing areas, of which 18 batches had species information (S1a Fig). 15 batches of samples (C21-C35) made of fake agarwood from the laboratory, mainly referring to the common counterfeiting methods in the market (S1b Fig). 18 samples of uncertain agarwood (C36-C53) collected from the trading market (S1c Fig). Such collections are permitted and legal. All voucher specimens of agarwood were kept in the herbarium of the agarwood Identification Center, Hainan Branch, Institute of Medicinal Plants, Chinese Academy of Medical Sciences.

**Table 1. Information on the samples used in this study.**

| **No.** | **Trade name** | **Genus/Species** | **Collected location** | **Type** |
| --- | --- | --- | --- | --- |
| **C01** | Bǎntóu agarwood ^#^ | *A. sinensis* | Haikou, China | The agarwood came from various producing areas. |
| **C02** | Agar-wit agarwood ^##^ | *A. sinensis* | Huazhou, China |  |
| **C03** | huǒlào agarwood ^##^ | *A. sinensis* | Maoming, China |  |
| **C04** | Chònglóu agarwood ^#^ | *A. sinensis* | Dingan, China |  |
| **C05** | píyǒu agarwood ^#^ | *A. sinensis* | Haikou, China |  |
| **C06** | kězǐ agarwood ^#^ | *A. crassna* | Hongkong, China |  |
| **C07** | Agar-wit agarwood ^##^ | *A. agallocha* | Bangladesh |  |
| **C08** | Wood chip ^##^ | *A..subintegra* | Laos |  |
| **C09** | Agar-wit agarwood ^##^ | *A. crassna* | Cambodia |  |
| **C10** | Wood chip | *A. crassna* | Vietnam |  |
| **C11** | Semi-submerged wood block | *Aquilaria* or *Gyrinops* | Sumatrina |  |
| **C12** | Wood block | *Aquilaria* or *Gyrinops* | Kalimantan |  |
| **C13** | Wood block | *Aquilaria* or *Gyrinops* | Papua New Guinea |  |
| **C14** | Special grade wood block | *A. filaria* | Ilian, Indonesia |  |
| **C15** | Wood chip | *A. malaccensis* | Malaysia |  |
| **C16** | Agar-wit agarwood ^##^ | *A. sinensis* | Huazhou, China |  |
| **C17** | Agar-wit agarwood ^##^ | *A. sinensis* | Haikou, China |  |
| **C18** | Wood chip | *A. yunnanensis* | Xishuangbanna, China |  |
| **C19** | Wood chip | *A. sinensis* | Zhangzhou, China |  |
| **C20** | Wood chip | *A. sinensis* | Qingshui, China |  |
| **C21** | Resin-free agarwood wood | *A. sinensis* | Haikou, China | The Laboratory made fake agarwood |
| **C22** | Ordinary agarwood A | *A. sinensis* | Haikou, China |  |
| **C23** | Ordinary agarwood B | *A. sinensis* | Haikou, China |  |
| **C24** | Resin-free agarwood wood 01 ^a)^ | *A. sinensis* | Haikou, China |  |
| **C25** | Resin-free agarwood wood 02 ^b)^ | *A. sinensis* | Haikou, China |  |
| **C26** | Resin-free agarwood wood 03 ^c)^ | *A. sinensis* | Haikou, China |  |
| **C27** | Resin-free agarwood wood 04 ^d)^ | *A. sinensis* | Haikou, China |  |
| **C28** | Ordinary agarwood A01 ^a)^ | *A. sinensis* | Haikou, China |  |
| **C29** | Ordinary agarwood A02 ^b)^ | *A. sinensis* | Haikou, China |  |
| **C30** | Ordinary agarwood A03 ^c)^ | *A. sinensis* | Haikou, China |  |
| **C31** | Ordinary agarwood A04 ^d)^ | *A. sinensis* | Haikou, China |  |
| **C32** | Ordinary agarwood B01 ^a)^ | *A. sinensis* | Haikou, China |  |
| **C33** | Ordinary agarwood B02 ^b)^ | *A. sinensis* | Haikou, China |  |
| **C34** | Ordinary agarwood B03 ^c)^ | *A. sinensis* | Haikou, China |  |
| **C35** | Ordinary agarwood B04 ^d)^ | *A. sinensis* | Haikou, China |  |
| **C36** | Rǔhú Chi-Nan agarwood | *A. sinensis* | Hannan, China | The uncertain agarwood from the market |
| **C37** | Tángjié Chi-Nan agarwood | *A. sinensis* | Hannan, China |  |
| **C38** | Aōshēn Chi-Nan agarwood | *A. sinensis* | Hannan, China |  |
| **C39** | Wood block | Unknown | Haikou, China |  |
| **C40** | Wood batten | Unknown | Bozhou, China |  |
| **C41** | Wood block | Unknown | Hehuachi, China |  |
| **C42** | Wood block | Unknown | Hehuachi, China |  |
| **C43** | Bracelet | Unknown | Vietnam |  |
| **C44** | Wood block | Unknown | Haikou, China |  |
| **C45** | Wood block | Unknown | Haikou, China |  |
| **C46** | Wood block | Unknown | Haikou, China |  |
| **C47** | Wood block | Unknown | United arab Emirates |  |
| **C48** | Wood chip | Unknown | Wanning, China |  |
| **C49** | Wood chip | Unknown | Haikou, China |  |
| **C50** | Wood block | Unknown | Dingan, China |  |
| **C51** | Wood block | Unknown | Bozhou, China |  |
| **C52** | Wood block | Unknown | Yulin, China |  |
| **C53** | Wood block | Unknown | Guangdong, China |  |

Note: ^#^ Wild agarwood, ^##^ Artificially induced agarwood.; a) aqueous extract solution of agarwood (AQE), b) alcoholic extract solution of agarwood (ALE), c) supercritical extract solution of agarwood (SUE), d) alcoholic extract solution with high resin content Chi-Nan agarwood (ALEH).

**Apparatus and reagents**

The features and image software were observed using a biological microscope (Nikon Eclipse 80i, Japan) fitted with a camera system (Nikon DS-5Mc, Japan). The tools included a water bath kettle (HH4, China), tissue softener (glycerin-ethanol, 1:1), chloral hydrate solution (Macklin, China), scalpel, and centrifugal tube.

**Treatment and observation**

We mimicked the steaming and oil injection methods that are commonly used on the market to produce fake agarwood. Usually, agarwood extracts, oils, or other substances (petroleum, spices, etc.) are mixed with resin-free agarwood wood or low-quality agarwood and counterfeited by steaming. The main steps of the laboratory-made fake agarwood were to place resin-free agarwood and ordinary agarwood in centrifuge tubes containing aqueous extract solution of agarwood (AQE), alcoholic extract solution of agarwood (ALE), supercritical extract solution of agarwood (SUE), and alcoholic extract solution with high resin content Chi-Nan agarwood (ALEH), respectively, and then steam-firing them for 12 hours at 90℃ in a water bath (S1a Fig). After cooling, the samples were cut into thin slices using the freehand slide-away slicing technique, while the samples after steaming in a water bath were then placed in centrifuge tubes containing water in a water bath at 70℃ for 0.5 hours and sliced using the same method (S1b Fig). After being divided into small pieces, the remaining 38 batches of samples were put in a centrifuge tube and heated to 70℃ for 0.5 hours. Using the freehand sectioning approach, thin slices were cut along the samples transection section. The thin section samples were placed in a chloral hydrate solution and examined using a biological microscope. With imaging software, the structural dimensions were measured.

**Results**

**Microstructural characteristics of genuine agarwood**

Twenty batches of genuine agarwood samples from various producing areas are shown in S1a Fig, and the microstructures of the transection section are shown in Fig 1. The resinous included phloem, and resinous rays and vessels were observed in all samples. The included phloem is diffuse, can be oblong, ribbon-shaped, or island-shaped, and is filled with brown or yellow-brown resin. The tangential and radial widths were 86.98-927.62 μm and 48.09-280 μm, respectively (Fig 2). Rays are radially extending, exclusively uniseriate, 5.1-62.07 μm wide, and uninterruptedly connected to the included phloem. Vessels are radially single or multiple, circular or ovoid, with tangential and radial widths of 11.04-223.77 μm and 4.11-162.11 μm, respectively. Although the size of the included phloem of agarwood is irregular and some samples (e.g., C6/ C7) are not completely filled with resin, it is present in all samples, and it is inferred that the resinous included phloem is a characteristic structure of agarwood.

**Fig 1.** **Microstructure of the** **transection section of the genuine agarwood from various producing areas.** C1-C20: sample number. IP: included phloem; R: ray; V: vessel. Scale bars 100 μm.

**Fig 2. The sizes of genuine agarwood included phloem, ray, and vessel.** A and B: the tangential and radial widths of the included phloem; C: ray width; D and E: the tangential and radial widths of the vessel.

**Microstructural characteristics of manufactured fake agarwood**

Although the resinous included phloem is characteristic structures of agarwood, it has not been verified whether the resins in these structures are naturally occurring or can be formed at a later stage by artificial addition. Although the resinous included phloem is characteristic of the structure of agarwood, it can be faked by using oil steaming and filling the extract. Therefore, this study used resin-free agarwood and ordinary agarwood to simulate the counterfeiting process by steaming the samples with the addition of agarwood extracts and observed the distribution of resin in the included phloem and its difference from the genuine agarwood. After steaming at 90℃ for 12 hours, the samples were all darkened in appearance to varying degrees, as shown in S1b Fig. The samples were sectioned, and the microstructures of the included phloem, rays, and vessels were all observed, as shown in Fig 3a. Compared to the control C21, C22 and C23, the samples did not have effective filling of resin in the included phloem and rays after treatment with the four agarwood extracts, especially in the resin-free agarwood (samples C24, C25, C26, and C27), which was more clearly observed. Meanwhile, the abovementioned samples were further subjected to a water bath at 70℃ for 0.5 h. The samples were further sectioned to observe the microstructure of the samples, as shown in Fig 3b. The resin in the included phloem and rays of ordinary agarwood A and B remains stable. Thus, the resin in the included phloem and rays of agarwood is naturally occurring and cannot be effectively accumulated even by the artificial addition of agarwood extracts.

**Fig 3. Microstructures of the transection section of the manufactured fake agarwood.** a. Samples steaming 90 ℃ for 12 hours. b. Samples after a treatment process followed by a water bath at 70℃ for 0.5 hours. C21-C35: Sample number; IP: included phloem; R: ray; V: vessel. Scale bars 200μm.

**Validation of microstructural characteristics of uncertain agarwood samples**

We observed the microstructures of 18 batches of uncertain samples from the market (Fig 4). After comparison with the genuine agarwood, it was revealed that the microstructures of 10 batches (C36, C37, C38, C39, C40, C42, C47, C51, C52, and C53) were consistent with that of agarwood, and the microstructures of 8 batches were not consistent with that of agarwood. The included phloem and rays of samples C36, C37, C38, and C39 were completely filled with resin, while the included phloem of samples C40, C42, and C51 were barely filled with resin, with only a small amount remaining around them. In addition, the included phloem of samples C47 and C52 were filled with resin but in sparse amounts. The microstructure of the remaining eight batches of samples differed from that of the agarwood, although in that they appeared to contain a brown substance. In addition, we soaked the remaining samples in water at room temperature. Interestingly, we found that the color of the soaking solution in the agarwood samples was pale yellow. However, the immersed solution colors of the uncertain samples C42 and C47 were yellow and reddish brown, respectively. The color of the immersed solution gradually deepened, accompanied by a pungent odor.

**Fig 4. The microstructure of the transection section of the uncertain agarwood.** C36-C53: Sample number. IP: included phloem; R: ray; V: vessel. Scale bars-100μm.

**Discussion**

**The resinous included phloem is a characteristic structure of genuine agarwood**

Wood identification is very important in the timber trade, fighting against illegal logging, wood certification, and forensic identification. Microanatomy sometimes provides information hidden by gross anatomy [36]. The xylem of *Aquilaria* is the most important structure, and it provides a place for resin formation and accumulation [31-32]. The included phloem and rays are interconnected to form a living parenchyma cell network in *A. sinensis* wood [33]. Therefore, verifying the prevalence of resinous included phloem in agarwood plays a decisive role in identifying the authenticity of agarwood.

In our study, we found that the microstructures of all genuine agarwood contained included phloem with a natural and regular distribution of brown or yellow-brown resin, although some samples had included phloem of different sizes. Gasson et al. report that all *Aquilaria* and *Gyrinops* are CITES listings and that the woods are anatomically quite similar [37-38]. To further verify this hypothesis, we searched for the presence of both structures in published species of the genera *Aquilaria* and *Gyrinops* and other genera of the family Thymelaeaceae. Among 12 species of *Aquilaria* and 4 species of *Gyrinops*, the wood contains included phloem (Table 2). Their microstructures are basically the same, which is consistent with the results of this paper. But the microstructures of the remaining species of the genera *Aquilaria* and *Gyrinops* have not been verified in the literature. Later studies are needed to further supplement the anatomical structures of these species in order to confirm that the included phloem is a characteristic structure of agarwood. In addition, the species of *Gonystylus* Teijsm. & Binn., *Aetoxylon* Airy Shaw, and *Phaleria* Jack do not included phloem in their xylem. However, there is no regularity in the size of the included phloem in different origins and species. This could potentially be used to differentiate between agarwood species and the time of agarwood formation, but was not explored in depth in this study.

It is worth noting that some species have also been reported to contain included phloem. This included phloem have the following characteristics: 1) The included phloem is very narrow, with only two to four cells per cell, such as noted in *Stylidium glandulosum* Salisb. (Stylidiaceae). 2) The included phloem is broad and roughly circular, such as seen in *Strychnos madagascariensis* Poir. (Loganiaceae). 3) The included phloem is crushed or stranded, such as noted in *Salvadora persica* L. (Salvadoraceae); 4) The included phloem is small and numerous in the early stage, with a short lifespan and a gradual decrease in number in the later stage, such as occurs in *Oenothera linifolia* Nutt. (Onagraceae) [39]. However, the microstructure of the included phloem is completely different from that of agarwood. Even though the appearance is similar to that of agarwood, counterfeit agarwood can be quickly identified using anatomy in this paper.

**Table 2. The wood structure of species capable of producing agarwood.**

| Species | Included phloem | References |
| --- | --- | --- |
| *A. agallochum* | + | [40]; This study |
| *A. beccariana* | + | [41] |
| *A. crassna* | + | [42]; This study |
| *A. cumingiana* | + | [41] |
| *A. filaria* | + | This study |
| *A. hirta* | + | [43] |
| *A. khasiana* | + | [44] |
| *A. malaccensis* | + | [32,42-43]; This study |
| *A. microcarpa* | + | [43,45] |
| *A. sinensis* | + | [11,31,33-34,42,46]; This study |
| *A. subintegra* | + | This study |
| *A. yunnanensis* | + | [47]; This study |
| *Gyrinops caudata* | + | [41] |
| *G. moluccana* | + | [41] |
| *G. versteegii* | + | [42,48-49] |
| *G. walla* | + | [50] |
| *Gonystylus affinis* | - | [12] |
| *G. bancanus* | **-** | [12] |
| *G. brunnescens* | **-** | [12] |
| *G.confuses* | **-** | [12] |
| *G. punctatus* | **-** | [37] |
| *G. macrophyllus* | **-** | [51] |
| *Aetoxylon sympetalum* | **-** | [52] |
| *Pbaleria* sp. | **-** | [41] |

Note: + Included phloem exist. - Included phloem does not exist.

**The resin in the included phloem and rays of agarwood is naturally occurring and cannot be effectively accumulated by artificial additions**

Agarwood is the resinous wood, and it is formed when the agarwood trees are damaged by physical force, insect, or fungal infection, and the resinous substance is embedded at the wound and gradually accumulates over time [12-15]. Due to the high price of agarwood, unscrupulous traders have been using artificial adulteration and substitution products to meet market demand and make staggering profits. It has been reported that the addition of illegal additives (carbon powder, rosin, extracts, colored oil, kerosene, etc.) can be used to increase the wood’s weight and color and create a resemblance to high-quality agarwood [3]. In our study, the fake samples were dark or black in color due to the counterfeiting of agarwood by the steaming method, and their appearance and smell were similar to those of genuine agarwood. However, by microscopic observation, the resin in the included phloem and rays of these counterfeited agarwood samples (resin-free agarwood wood and ordinary agarwood) did not accumulate effectively.

The included phloem of genuine agarwood contains resin. It was found that the resin in the included phloem of some of the agarwood samples was not completely filled, a phenomenon that may be related to the short time of agarwood formation, the unstable method of induction, or incomplete primary processing. In general, the included phloem and rays of agarwood are supposed to contain resin.

Our validation experiments also confirmed that there was no significant loss or reduction of resin in the included phloem and rays of the genuine agarwood under the same steaming conditions. It has also been shown that when agarwood is immersed in water, the resin dissolves very slowly, and the resin remains stable for a certain period of time [53]. In addition, we found that the color of the soaking water solution in the genuine agarwood samples was pale yellow. However, the soaking water solution colors of 2 batch uncertain samples were yellow and reddish brown, respectively. The color of the immersed solution gradually deepened, accompanied by a pungent odor. Therefore, we conclude that additives have been added to the agarwood.

**Searching for resinous included phloem under the microscope provides a convenient and rapid method for the authentication of agarwood**

We selected included phloem as important elements to identify in all samples of agarwood. Meanwhile, the reliability of this method was validated by the identification of authentic and uncertain agarwood from the market and the fake agarwood. A standard operation procedure was formed, as illustrated in Fig 5, which first described the appearance trait of the samples, followed by basic sample processing, including sampling, warm bath, permeabilization, and sealing; finally, the included phloem and ray structures containing resin were observed under the microscope to determine the authenticity of the samples. Microstructure identification provides reliable results compared to the trait identification of agarwood. In addition, compared with chemical analysis and molecular identification, the microstructure identification method is simple, low cost, and does not require expensive and sophisticated equipment. The microstructure identification method used in this study was evaluated in terms of equipment, testing time, cost, technicians, and reliability of results and it was able to identify the authenticity of agarwood quickly, conveniently, and accurately ( S1 Table).

**Fig 5. Standard operating procedures for** **microstructure identification of agarwood.**

**Conclusion**

In this study, by comparing the authenticity of the microstructures of agarwood from different origins and artificially simulated fake agarwood, it was found that the included phloem of genuine agarwood was filled with brown to brownish resins, which were also deposited in the rays, naturally distributed, and stably present. The filling of resin in the included phloem of agarwood did not occur after artificially simulated counterfeiting treatments. Fake agarwood is divided into two categories, one itself is wood confusion (without included phloem), and the other is artificially counterfeiting (there is included phloem, but it does not contain resin, or counterfeiting filler material can be dissolved out). Therefore, the resinous included phloem is an important characteristic structure of agarwood and can be used as a basis for authenticity identification. This will provide a convenient and rapid method for the promotion and popularization of agarwood authentication in trade, customs enforcement, CITES management, and other fields.

**Supporting information**

**S1 Fig. Experimental samples used in this study.** a. The agarwood came from various producing areas. b. Laboratory-made fake agarwood. Resin-free agarwood (C21), ordinary agarwood A (C22) and ordinary agarwood B (C23) were soaked in four agarwood extracts and steamed for 12 hours in a 90℃ water bath, followed by 0.5 hours in a 70℃ water bath. After cooling, thin slices were cut in the transection section direction of the samples treated in both stages. c. Uncertain agarwood from the market. AQE is an aqueous extract of agarwood. ALE is an alcoholic extract of agarwood. SUE is a supercritical extract of agarwood. ALEH is an alcoholic extract with a high resin content (Chi-Nan) of agarwood. WAB is water. C1-C53: Sample number. (tif.)

**S1 Table. Comparison of the results of this study with existing techniques for the identification of agarwood.** (docx.)

**Acknowledgments**

We thank the agarwood Identification Center, Hainan Branch, Institute of Medicinal Plants, Chinese Academy of Medical Sciences and Peking Union Medical College for supporting the collection of samples and microstructure experiments in this study.

**Author contributions**

**Conceptualization:** Yangyang Liu, Jianhe Wei.

**Data curation:** Jian Feng, Anzhen Xie.

**Formal analysis:** Jian Feng.

**Investigation:** Jian Feng, Yun Yang, Peiwei Liu.

**Methodology:** Jian Feng, Yangyang Liu, Peiwei Liu, Jianhe Wei.

**Resources:** Yangyang Liu, Jianhe Wei, Jian Feng.

**Validation:** Jian Feng, Peiwei Liu, Anzhen Xie.

**Visualization:** Jian Feng.

**Writing – original draft:** Jian Feng.

**Writing – review & editing:** Jian Feng, Yangyang Liu, Jianhe Wei.

**Conflict of interest**

The authors have declared that no competing interests exist.

**Data availability statement**

All the data generated and analyzed during this study are included in this article, and any additional data can be requested on request from the corresponding author.

**References**

1. Hashim YZH, Kerr PG, Abbas P, Salleh HM. *Aquilaria* spp. (agarwood) as source of health beneficial compounds: A review of traditional use, phytochemistry and pharmacology. J Ethnopharmacol. 2016; 189: 331-360. https://doi.org/10.1016/j.jep.2016.06.055.
2. Liu YY, Wei JH, Gao ZH, Lyu JC. A review of quality assessment and grading for agarwood. Chin Herbal Med. 2017; 9: 22-30. https://doi.org/10.1016/S1674-6384(17)60072-8.
3. Barden A, Anak NA, Mulliken T, Song M. Heart of the matter: agarwood use and trade and CITES implementation for *Aquilaria malaccensis*. TRAFFIC International, Cambridge; 2000.
4. Chitre T, Bhutada P, Nandakumar K, Somani R. Miniyar P, Mundhada Y, et al. Analgesic and anti-inflammatory activity of heartwood of *Aquilaria agallocha* in laboratory animals. Pharmacol online. 2007; 1: 288-298.
5. Rahman H, Eswaraiah MC, Dutta AM. Anti-arthritic activity of leaves and oil of *Aquilaria agallocha*. Haya: Saudi J life sci. 2016; 1: 34–43.
6. Takemoto H, Ito M, Shiraki T, Yagura T, Honda G. Sedative effects of vapor inhalation of agarwood oil and spikenard extract and identification of their active components. J Nat Med. 2008; 62: 41-46. https://doi.org/10.1007/s11418-007-0177-0.
7. Lee SY, Mohamed R. The Origin and Domestication of *Aquilaria*, an Important Agarwood-Producing Genus. In: MOHAMED R. (eds) agarwood. Tropical Forestry. Springer, Singapore; 2016. https://doi.org/10.1007/978-981-10-0833-7_1.
8. Liu J. Global distribution of incense resources and the current status of research on the method of agarwood formation. China food drug admin mag. 2020; 12: 100-104. https://doi.org/10.3969/j.issn.1673-5390.2020.12.015.
9. Wyn LT, Anak NA. Wood for the trees: a review of the agarwood (gaharu) trade in Malaysia. TRAFFIC Southeast Asia, Malaysia; 2010.
10. Convention on International Trade in Endangered Species of Wild Fauna and Flora (CITES). Thirteenth meeting of the Conference of the Parties, Bangkok; 2004.
11. Yin Y, Jiao L, Dong M, Jiang X, Zhang S. Wood Resources, Identification, and Utilization of Agarwood in China. In: MOHAMED R. (eds) agarwood. Tropical Forestry. Springer, Singapore; 2016. https://doi.org/10.1007/978-981-10-0833-7_2.
12. Nobuchi T, Siripatanadilok S. Preliminary observation of *Aquilaria crassna* wood associated with the formation of aloeswood. Bull Kyoto Univ Forests. 1991; 63: 226-235.
13. Liu YY, Chen HQ, Yang Y, Zhang Z, Wei JH, Meng H, et al. Whole-tree agarwood-inducing technique: An efficient novel technique for producing high-quality agarwood in cultivated *Aquilaria sinensis* trees. Molecules, 2013; 18: 3086-3106. https://doi.org/10.3390/molecules18033086.
14. Kalita J, Bhattacharyya PR, Boruah HPD, Unni BG, Lekhak H, Nath SC. Association of *Zeuzera conferta* Walker on agarwood formation in *Aquilaria malaccensis* Lamk. Asian J Plant Sci Res. 2015; 5: 4-9.
15. Chen XY, Sui C, Liu YY, Yang Y, Liu PW, Zhang Z, et al. Agarwood formation induced by fermentation liquid of *Lasiodiplodia theobromae*, the dominating fungus in wounded wood of *Aquilaria sinensis*. Curr Microbiol. 2017; 74: 460-468. https://doi.org/10.1007/s00284-016-1193-7.
16. Persoon GA, van Beek HH. Growing ‘The Wood of The Gods’: Agarwood Production in Southeast Asia. In: Snelder, D.J., Lasco, R.D. (eds) Smallholder Tree Growing for Rural Development and Environmental Services. Advances in Agroforestry, vol 5. Springer, Dordrecht; 2008. https://doi.org/10.1007/978-1-4020-8261-0_12.
17. Jim CY. Cross-border itinerant poaching of agarwood in Hong Kong’s peri-urban forests. Urban For Urban Green. 2015; 14: 420-432. https://doi.org/10.1016/j.ufug.2015.04.007.
18. Xu YH, Zhang Z, Wang MX, Wei JH, Chen HJ, Gao ZH, et al. Identification of genes related to agarwood formation: transcriptome analysis of healthy and wounded tissues of *Aquilaria sinensis*. BMC genomics. 2013; 14: 1-16. https://doi.org/10.1186/1471-2164-14-227.
19. Tan CS, Isa NM, Ismail I, Zainal Z. Agarwood induction: current developments and future perspectives. Front plant sci. 2019; 10: 122. https://doi.org/10.3389/fpls.2019.00122.
20. Lin YX, Feng TT, Dai JP, Liu QZ, Cai YM, Kuang JR, et al. DNA barcoding identification of IUCN Red listed threatened species in the genus *Aquilaria* (Thymelaeaceae) using machine learning approaches. Phytochem Lett. 2023; 55: 105-111. https://doi.org/10.1016/j.phytol.2023.04.007.
21. Yang ZH, Fang HB, Tao CT, Jiao YB, Cheng YX. Eight new 2-(2-phenylethyl) chromone derivatives from agarwood of *Aquilaria sinensis* with anti-inflammatory activity. Fitoterapia. 2023; 169: 105564. https://doi.org/10.1016/j.fitote.2023.105564.
22. Espinoza EO, Lancaster CA, Kreitals NM, Hata M, Cody RB, Blanchette RA. Distinguishing wild from cultivated agarwood (*Aquilaria* spp.) using direct analysis in real time and time of-flight mass spectrometry. Rapid Commun Mass Spectrom. 2014; 28: 281-289. https://doi.org/10.1002/rcm.6779.
23. Yu Z, Dong WH, Wang YL, Li W, Guo ZY, Mei WL, et al. Identification of aroma‐active components from cultivated agarwood ‘Qi-Nan’based on GC-O-MS combined with aroma extract dilution analysis. Flavour and Fragrance Journal, 2023; 38: 392-403. https://doi.org/10.1002/ffj.3754.
24. Du Z, Wang HJ, Li XL, Dong MY, Chi BQ, Tian ZH, et al. Rapid screening and characterization of 2-(2-phenylethyl) chromones in agarwood by UHPLC-Q-Exactive Orbitrap-MS. Food Chem. 2023; 424: 136400. https://doi.org/10.1016/j.foodchem.2023.136400.
25. Wang CH, Peng DQ, Liu YY, Wu YL, Guo P, Wei JH. Agarwood alcohol extract protects against gastric ulcer by inhibiting oxidation and inflammation. Evidence-based Complementary and Alternative Medicine. 2021; 9944685. https://doi.org/10.1155/2021/9944685.
26. Gogoi R, Sarma N, Begum T, Chanda SK, Lekhak H, Sastry GN, et al. Agarwood (*Aquilaria malaccensis* L.) a quality fragrant and medicinally significant plant based essential oil with pharmacological potentials and genotoxicity. Ind Crop Prod. 2023; 197: 116535. https://doi.org/10.1016/j.indcrop.2023.116535.
27. Chinese Pharmacopoeia Commission. Pharmacopoeia of the people´s republic of China, Volume I. China Med Sci Tech Press; 2020.
28. Youssef J, Döll-Boscardin PM, Farago PV, Duarta MR, Budel JM. Gochnatia polymorpha: macro- and microscopic identification of leaf and stem for pharmacognostic quality control. Rev Bras de Farmacogn. 2013; 23: 588-591. https://doi.org/10.1590/S0102-695X2013005000054.
29. Yuan YD, Liu X, Wang JP, Zhang JC. Morphological and microscopic identification of three major medicinal *Dendrobium* species in Ta-pieh Mountains area. Microsc Res Techniq. 2019; 82: 483-493. https://doi.org/10.1002/jemt.23191.
30. Wheeler EA, Gasson PE, Baas P. Using The Inside Wood Web Site: Potentials And Pitfalls. IAWA J. 2020; 41: 412-462. http://dx.doi.org/10.1163/22941932-bja10032.
31. Zhang XL, Liu YY, Chen HJ, Xu YH, Zhang Z, Yang Y, et al. Study on xylem structure and histochemistry in *Aquilaria sinensis*. J Shangdong Univ (Nat Sci). 2012; 47: 1-5. https://doi.org/10.6040 /j.issn.1671-9352.2012.07.001.
32. Mohamed R, Wong MT, Halis R. Microscopic Observation of ‘Gaharu’ Wood from *Aquilaria malaccensis*. Pertanika J Trop Agric Sci. 2013; 36: 43-50.
33. Liu PW, Zhang XL, Yang Y, Sui C, Xu YH, Wei JH. Interxylary phloem and xylem rays are the structural foundation of agarwood resin formation in the stems of *Aquilaria sinensis*. Trees, 2019; 33: 533-542. https://doi.org/10.1007/s00468-018-1799-4.
34. Liu PW, Zhang YX, Yang Y, Lv FF, Wei JH. Programmed cell death might involve in progress of wounding induced agarwood formation in stems of *Aquilaria sinensis*. Microsc Res Techniq. 2022; 1-9. https://doi.org/10.1002/jemt.24140.
35. Zhang Y, Meng H, Lyu FF, Fan XH, Liu PW, He X, et al. Temporal characteristics of agarwood formation in *Aquilaria sinensis* after applying whole-tree agarwood-inducing technique. Chinese Herbal Medicines. 2023; 15: 37-44. https://doi.org/10.1016/j.chmed.2022.07.003.
36. Timell. Comparative wood anatomy. Springer Berlin; 2001.
37. Gasson P, Warner K, Lewis GP. Wood anatomy of *Caesalpinia* s.l.: *Caesalpinia* s.s., *Coulteria*, *Erythrostemon*, *Guilandina*, *Libidibia*, *Mezoneuron*, *Poincianella* and *Tara* (Leguminosae, Caesalpinioideae, Caesalpinieae). IAWA J. 2009; 30: 247-276. https://doi.org/10.1163/22941932-90000218.
38. Gasson P, Baas P, Wheeler E. Wood anatomy of CITES-Listed tree species. IAWA J. 2011; 32: 155-198. https://doi.org/10.1163/22941932-90000050.
39. Carlquist S. Interxylary phloem: Diversity and functions. Brittonia. 2013; 65: 477-495. https://doi.org/10.1007/s12228-012-9298-1.
40. Kim IR, Park SJ. Anatomical features of agarwood xylem. Kor J Herbology. 2005; 20: 9-18.
41. Andianto A. Ciri anatomi lima jenis kayu penghasil gaharu dan dua jenis kerabatnya. Jur Pen Hasil Hutan. 2010; 28: 169-183.
42. Luo B, Imai T, Sugiyama J, Maroem SN, Mulyaningsih T, Itoh T. The occurrence and structure of radial sieve tubes in the secondary xylem of *Aquilaria* and *Gyrinops*. IAWA J. 2019; 1-16. https://doi.org/10.1163/22941932-00002013.
43. Nordahlia AS, Lim SC, Anwar UMK. Wood anatomical features of *Aquilaria* (Thymelaeaceae) and *Gonystylus* (Gonystylaceae) in Malaysia. Malayan Nat J. 2017; 69: 63-69.
44. Yang Y, Feng J, Zhu JX, Liu YY, Chen DL, Wei JH. Quality of agarwood produced by whole tree agarwood-inducing technique (Agar-Wit) from China and Southeast Asian countries. Chin Pharm J. 2019; 54: 1988-1994. https://doi.org/10.11669/cpj.2019.23.014.
45. Saikia N, Nath SC. Anatomical Characters of few Infraspecific Variants of *Aquilaria malaccensis* Lamk. Bull Bot Surv India. 2004; 46: 468-479.
46. Feng J, Hou WC, Chen L, Chen XQ, Yang Y, Liu YY, et al. Quality analysis and evaluation of agarwood produced by "Chi-Nan" germplasm based on standard of Chinese Pharmacopoeia. Mod Chin Med. 2022; 24: 432-437.
47. Su J, Liu Z, Li RC, Ren CQ, Yang XJ. Preliminary Study on Timber Structure of *Aquilaria yunnanensis*. Chin J Trop Agric. 2016; 36: 30-34. https://doi.org/10.12008/j.issn.1009-2196.2016.04.007.
48. Tabata Y, Widjaja E, Mulyaningsih T, Parman I, Wiriadinata H, Mandang YI, et al. Structural survey and artificial induction of aloeswood. Wood Research: Bull Wood Res Inst Kyoto Univ. 2003; 90: 11-12.
49. Hidayat W, Mulyaningsih T, Muspiah A. Agarwood formation in *Gyrinops versteegii* seedlingstage using four types of inducers. Jur Sylva Lestari. 2022; 10: 233-243. https://doi.org/10.23960/jsl.v10i2.523.
50. Subasinghe SMCUP, Hettiarachchi DS. Agarwood resin production and resin quality of *Gyrinops walla* Gaertn. Int J Agric Sci. 2013; 3: 357-362.
51. Scurfield G, Silva SR, Ingle HD. Vessel wall structure: An investigation using scanning electron microscopy. Aust J Bot. 1970; 18: 301-312. https://doi.org/10.1071/BT9700301.
52. Yan ZC, Chen Y, Zhou C. Research on *Aetoxylon sympetalum* wood of Thymelaeaceae. Qual Tech Superv Res. 2019; 2: 27-29. https://doi.org/10.15902/j.cnki.zljsjdyj.2019.02.007.
53. Jok VA, Radzi NC, Hamid KHK. Agarwood oil yield as a result of changes in cell morphology due to soaking process. Procedia Soc Behav Sci. 2015; 195: 2443-2450. https://doi.org/10.1016/j.sbspro.2015.06.387.
