# Supplementary material for: Resinous included phloem as a key indicator of authentic or fake agarwood
Source: PLoS One. 2024 Dec 2;19(12):e0312102. doi: 10.1371/journal.pone.0312102 (PMC11611195; doi:10.1371/journal.pone.0312102)
Supplement: Attachment — Submitted filename: Response to Reviewers1+2.docx [file pone.0312102.s005.docx]

**Point-by-point response to** **reviewer 1**

**Reply to journal requirements**

We are very grateful to the reviewer for the specification of this paper, and we have made the required article-by-article changes as detailed below:

**Q1. The language needs improvement.**

**Answer:** Thank you very much for your review. The language of the manuscript has been revised and embellished.

**Q2. The supplementary information can form a part of the main text.**

**Answer:** Thank you very much for your review. Changes have been made in response to your comments, and S1a Fig, S1b Fig, S1c Fig, and S1 Table have been added to the manuscript. To avoid duplication, Table 1 has been placed in the annexed material as S1 Table. Revisions to other elements have been identified in the manuscript with yellow underlining.

**Q3. Data related to the anatomical characteristics and be provided as supplementary information. T****he sectionwise comments are provided as attachment.**

**Answer:** Thank you very much for your review. In response to your comments, we have made some changes and have provided the data charts for anatomical features as an attachment, and other content changes are identified with a yellow underline.

**Q4.** **Title:** Could be modified as 1) Assessing Authenticity in Agarwood through the Presence of Resinous Included Phloem; 2) Resinous Included Phloem as a Key Indicator of fake or authentic Agarwood.

**Answer:** Thank you very much for your review. We have chosen your point 2 as a more appropriate title for the article and have revised it in the manuscript.

**Q5.** **Introduction:** **1)** Language needs improvement; **2)** A logical flow is lacking in the introduction. It fails to introduce the need for identifying included phloem characterisitics to establish a reliable method for identifying authentic agarwood.

**Answer:** Thank you very much for your review. We have made changes to the introduction.

**Q6.** **Materials and Methods:** **1)** Clarify the terms "uncertain agarwood", " producing areas " to ensure it's easily understood by readers. **2)** What does ‘mainly referring to the common counterfeiting methods in the market’ mean? **3)** When you mention 53 batches, how many samples were present in each batch? Or do you mean 53 samples? **4)** Ensure consistency in terminology. Retain either batch or sample. **5)** How many observations were recorded per sample? **6)** What statistical analysis was employed? **7)** Overall, the essential details of the sample collection process is conveyed, but breaking down the information and providing clarifications can enhance readability and comprehension.

**Answer:** Thank you very much for your review. We have revised and clarified the seven issues you raised in the manuscript.

**Q7. Results:** The results effectively communicate the findings, but there are areas where clarity and conciseness could be improved.

**Answer:** Thank you very much for your review. We have followed the changes made to the results section.

**Q8. Discussion:** **1)** The readability of the discussion is moderate. While the information presented is clear in most parts, there are areas where the complexity of the subject matter or the improper usage of grammar poses a challenge. **2)** With minor revisions to simplify the language and complex concepts, the readability of the report could be improved. **3)** Consider rephrasing or condensing repetitive information to avoid redundancy.

**Answer:** Thank you very much for your review. We have made the changes you suggested.

**Point-by-point response to reviewer 2**

**Reply to journal requirements**

We are very grateful to reviewer for the specification of this paper, and we have made the required article-by-article changes as detailed below:

**Q1.** **The manuscript is very flimsy in its scientific content and poor in language. It needs a major revision before being considered further for evaluation.**

**Answer:** Thank you very much for your review. The language and content of the manuscript has been revised and embellished.

**Q2. The authors have included many paragraphs that are irrelevant; it seems like they were included just to increase the volume of the text.**

**Answer:** Thank you very much for your review. The contents of the manuscript have been verified and revised.

**Q3. The details of any statistical analysis conducted are not clear.**

**Answer:** Thank you very much for your review. Sample observation and statistical analysis methods have been added to the Materials and Methods section.

**Q4. Is the repeatability of the experiments doubtful?**

**Answer:** Thank you very much for your review. We firmly believe that the results of the manuscript are reliable. We analyzed the samples of authentic agarwood from different producing areas by observation, and at least 12 replicates of thin slices of each sample were extracted for microstructure observation and analysis. Methodological validation was also carried out on artificially fake agarwood, and the resin-containing included phloem was found to be the characteristic structure of authentic agarwood. In addition, our team specializes in the research of authentication and quality control of agarwood and has established the China's first agarwood authentication center, which is also confirmed by the CMA accreditation issued by the Chinese government. The methods used in this research are simple and practical and meet the use of agarwood-related industries.

**Q5. The title of the manuscript is inappropriate and does not spell out the objective of the study clearly.**

**Answer:** Thank you very much for your review. We chose the appropriate title “Resinous included phloem as a key indicator of authentic or fake agarwood” and revised it in the original manuscript.

**Q6. The manuscript contains many typographical errors.**

**Answer:** Thank you very much for your review. Changes have been checked and corrected in the manuscript.

**Q7. The plagiarism of the manuscript may also be checked.**

**Answer:** Thank you very much for your review. The data in our manuscript is authentic and reliable and there is no plagiarism. our team specializes in the research of authentication and quality control of agarwood and has established the China's first agarwood authentication center, which is also confirmed by the CMA accreditation issued by the Chinese government. The methods used in this research are simple and practical and meet the use of agarwood-related industries. The experimental data in the manuscripts involved are authentic.
